# The Revolution in Human Monogenic Disease Mapping

**DOI:** 10.3390/genes5030792

**Published:** 2014-09-05

**Authors:** Emma Duncan, Matthew Brown, Eileen M. Shore

**Affiliations:** 1 University of Queensland Diamantina Institute, Translational Research Institute, Princess Alexandra Hospital, 37 Kent Road, Woolloongabba, Brisbane 4102, Queensland, Australia; E-Mails: emma.duncan@uq.edu.au (E.D.); matt.brown@uq.edu.au (M.B.); 2 Department of Endocrinology and Diabetes, Royal Brisbane and Women’s Hospital, Herston 4029, Queensland, Australia; 3 Center for Research in FOP and Related Disorders, Department of Orthopaedic Surgery, Perelman School of Medicine, University of Pennsylvania, 3450 Hamilton Walk, Philadelphia, PA 19104, USA; 4 Department of Genetics, Perelman School of Medicine, University of Pennsylvania, Curie Boulevard, Philadelphia, PA 19104, USA

**Keywords:** Human Genome Project, fibrodysplasia ossificans progressiva, monogenic diseases, disease gene discovery, NGS

## Abstract

The successful completion of the Human Genome Project (HGP) was an unprecedented scientific advance that has become an invaluable resource in the search for genes that cause monogenic and common (polygenic) diseases. Prior to the HGP, linkage analysis had successfully mapped many disease genes for monogenic disorders; however, the limitations of this approach were particularly evident for identifying causative genes in rare genetic disorders affecting lifespan and/or reproductive fitness, such as skeletal dysplasias. In this review, we illustrate the challenges of mapping disease genes in such conditions through the ultra-rare disorder fibrodysplasia ossificans progressiva (FOP) and we discuss the advances that are being made through current massively parallel (“next generation”) sequencing (MPS) technologies.

## 1. Introduction

Until very recently, mapping the causative gene for monogenic diseases depended on finding families with demonstrable Mendelian inheritance of the disease, preferably in multiple generations. Linkage approaches in such families were successful in identifying mutations responsible for many of the more frequent monogenic diseases and traits [1]. However, identifying the underlying mutations in rare genetic diseases, especially those associated with low reproductive fitness, late onset diseases, or diseases with early lethality, proved much more challenging.

The Human Genome Project (HGP) set the stage for success in meeting these challenges. The ultra-rare disorder fibrodysplasia ossificans progressiva (FOP), with a frequency of one in two million, is an example of such a success. Linkage in FOP families identified chromosomal regions of interest; Human Genome Project databases then facilitated the identification of candidate genes within the linkage region and permitted the efficient identification of altered DNA sequences. FOP was found to be caused by a recurrent single nucleotide substitution occurring in >95% of patients. Despite the ultimate success in mapping the FOP gene, this discovery took decades of effort to identify families with inheritance of the disease (linkage analysis was eventually accomplished with just five two-generation families) followed by the time-consuming tasks of conducting genome-wide linkage analysis, and subsequent identification and re-sequencing of candidate genes within the linkage intervals. Knowing the genetic mutation in FOP has led quickly to better understanding of the underlying pathology and directed strategies for treatments, neither of which were possible before molecular aetiology was determined.

Building on the technology, computation, and scientific information generated through the HGP, the continued advances in mapping disease genes have been extraordinarily rapid. Faced today with the challenge of identifying a rare gene mutation in a disease like FOP, high-throughput exome and/or whole genome sequencing approaches would identify the genetic mutation rapidly. Moreover, gene identification may require sequencing of only a small number of unrelated cases, small families, or, in some cases, even a single proband. These breakthroughs are leading to an upsurge in disease gene discoveries with their associated benefits [2].

After mapping a disease-causing gene, many challenges remain in understanding additional genetic contributions to disease onset and progression. In FOP, for example, although the main disease characteristics are unique and readily recognized there is significant variability in the age of disease onset, the rate of disease progression, and the severity of disease, likely to arise from as yet un-identified genetic causes. Similar issues exist with many common heritable diseases such as osteogenesis imperfecta and Marfan’s syndrome. With further development of genome technologies, the ability to understand phenotypic variability and the participation of genetic modifiers is becoming a reality.

## 2. Linkage Mapping

### 2.1. History of Linkage Mapping

At the turn of the twentieth century, Archibald Garrod coined the term “inborn errors of metabolism” to explain the increased incidence of alkaptonuria (and, subsequently, also cystinuria, pentosuria, and albinism) in consanguineous families compared with the general population, and suggested that these conditions were caused by transmissible elements within families [3]. Since this time, the challenge has been to identify these errors.

For most of the twentieth century, linkage mapping was the standard means of identifying the gene/s underlying an inherited disorder. Linkage is the co-segregation of a genetic region with disease phenotype within a family. Markers, such as restriction fragment length polymorphisms, microsatellites, or single nucleotide polymorphisms (SNPs), are genotyped in family members. Markers close to a disease-causing mutation will be co-inherited with the disease-causing mutation, unless separated by a meiotic event—the closer the marker to the disease-causing gene, the less likely it will be separated at meiosis. An area of linkage within a family may thus extend a considerable genetic distance. Whole genome linkage scans, in which approximately 300–400 microsatellite markers are genotyped in family members, usually result in identification of regions of linkage spanning 10–20 cM (on average ~10–20 million DNA bases). Such a region may harbor many hundreds of genes, and fine mapping (by further marker genotyping and/or sequencing of candidate genes) will usually be necessary to identify the exact causative gene.

### 2.2. Weaknesses of the Linkage Approach

Traditional “parametric” linkage analysis compares the likelihood of the observed transmission of genetic markers in relation to the trait or disease, in the context of a specified model of inheritance. Non-parametric methods not requiring knowledge of mode of inheritance can be used, though are less powerful when the correct mode of inheritance is known. Diseases with late onset of clinical features or with incomplete penetrance are harder to map by linkage due to possible incorrect attribution of disease status among family members. Diseases with significant gene/environment interaction present similar issues, unless all family members are exposed to the same environmental stressors. The ability to map a gene also depends on the number of informative meioses within a pedigree. Thus, large families with many affected individuals—especially distantly-related individuals who will have a high number of meioses and recombination events between them—are the most useful for linkage mapping. Diseases that affect reproductive fitness (such as skeletal dysplasias) are less likely to have such large informative pedigrees. One solution to this problem is to use many families affected by the same condition in order to identify a common linkage region shared by affected persons within each family. This strategy requires that the disease be caused by mutations in the same single common locus in all families, although the causative mutation within this common locus may be unique in each individual family. The approach will, therefore, not be successful for diseases with “phenocopies”, in which a clinical phenotype may arise from mutations in more than one gene. (For example, phenocopies might result if mutations in various genes along a biological pathway resulted in a common phenotypic endpoint.) “Pooling” linkage information from families with disparate underlying causes would not be a successful strategy. Extremely rare diseases are, by definition, extremely rare; obtaining sufficient number of families to pool their linkage information will usually require international collaboration. Lastly, novel/spontaneous mutations (those newly occurring within an individual) cannot be mapped by linkage.

### 2.3. Successes in Linkage Mapping: Monogenic vs. Complex Diseases

Despite these limitations, mapping monogenic diseases by linkage has been quite successful, even for rare diseases, with well over 1000 monogenic (Mendelian) disorders mapped by the turn of the century [1]. Approximately two-thirds of the 400 or so recognized skeletal dysplasias were mapped by linkage or similar approaches by 2010 [4]. In contrast, mapping complex diseases by linkage was much less successful. Complex genetic diseases are typically polygenic in nature: affected individuals have different variants in multiple, but overlapping, sets of genes, with each variant contributing only a small part to the final overall phenotype. Before the era of high throughput microarray genotyping and the advent of genome-wide association studies (GWAS), only a handful of genes had been identified for complex diseases using linkage [5].

## 3. Mapping the FOP Gene Highlights the Challenges

Fibrodysplasia ossificans progressiva (FOP; MIM 135100) is a severe disorder of progressive and extensive extra-skeletal ossification. Heterotopic ossification in FOP begins in childhood within connective tissues, such as skeletal muscle, tendon, and ligament. Onset of bone formation can be induced by trauma, or may occur spontaneously. Bone formation is episodic, leading to cumulative disability and shortened lifespan [6].

Reproductive fitness is low in FOP, resulting in infrequent inheritance and a population frequency (about one per two million) that reflects the rate of new mutations. When the search for the FOP gene began, only very few cases of familial inheritance of FOP had been reported, with most known cases occurring *de novo* in families (reviewed in [7,8]). Although these few family pedigrees suggested an autosomal dominant mode of inheritance, the paucity of families with transmission of FOP made genome-wide linkage analysis, the state-of-the-art approach at the time, an impractical strategy for gene identification.

Eventually, four small pedigrees with autosomal dominant inheritance of FOP were identified, although some had ambiguous clinical features. An initial genome-wide linkage analysis using 240 microsatellite markers (spaced ≤ 25 cM) was conducted, although it was recognized that there was incomplete/uneven marker coverage across the genome and many markers lacked sufficient informativity [9]. Whilst the initial linkage analysis focused attention on a chromosome 4 locus [9], further analysis revealed additional linked loci on chromosomes 2 and 6. However, subsequent sequencing analysis of numerous candidate genes in the linkage regions revealed no identifiable mutations. The limited information available at this time about the human genome, including gene locations and sequence, made this process much more challenging.

As genome analysis technologies continued to develop and additional families with autosomal dominant transmission of FOP were identified, further genome-wide linkage studies were performed. These used both a higher density SNP marker panel as well as more dense and informative microsatellite panels, which were combined in a single analysis. The international team involved focused their studies on five two-generation families with stringent and unambiguous phenotypic features of FOP in all affected family members (congenital malformation of the great toes and progressive heterotopic ossification in characteristic anatomic patterns). Consistent linkage was then demonstrated with the chromosome 2q23–24 interval in all five families (LOD score 2.3) [8], with no other locus segregating with the disease in all pedigrees. Using better characterized and annotated human DNA sequences generated through the Human Genome Project (HGP), we selected candidate genes within the 16.5 Mb linkage interval for sequencing and mutation identification. The interval contained more than 40 known genes, however, concurrent investigations had identified the BMP signaling pathway as altered in FOP [10,11,12,13], and genes in this pathway were given high priority. One such gene was the *Activin A type I receptor* gene (*ACVR1*; OMIM 102576; also known as *Alk2* or *ActRI*), encoding a receptor for bone morphogenetic protein (BMP).

DNA sequence analysis of all *ACVR1* protein-coding exons and splice junctions identified a heterozygous c.617G>A (Arg206His; CGC ≥ CAC) mutation present in all affected members in these FOP families, with the same mutation present in multiple sporadic cases of FOP [8].

Cumulative data over the past eight years shows that FOP is caused by this recurrent single nucleotide substitution in >95% of patients ([14,15,16]). In exceptional cases of FOP, mainly those whose phenotype varies slightly from the description above, affected individuals have mutations at other amino acid positions in *ACVR1* in the glycine-serine (GS) or protein kinase domains [14,17]. Thus far, all patients clinically diagnosed with the “classical” FOP phenotype have *ACVR1* mutations, and these mutations are fully penetrant.

## 4. Massive Parallel Sequencing: A New Era

The mapping of rare monogenic disorders has been completely transformed by the advent of massive parallel sequencing (MPS), also known as “next-generation” sequencing. In the last few years, the causative genes for dozens of monogenic disorders have been identified using MPS, and the rate of discovery has been exponential. To illustrate this latter point, we recently published a review of MPS in skeletal dysplasias; at the time of paper submission (April 2013) 26 papers had been published using MPS to identify the causative gene for 22 skeletal dysplasias; by the time of paper acceptance (July 2013) a further ten papers had added another six novel skeletal dysplasia genes to the list [2]. Since then, further causative genes for skeletal dysplasias, as well as a host of other Mendelian disorders, have been identified, and it is likely that many of the remaining unmapped monogenic diseases will prove tractable to mapping by MPS.

### 4.1. MPS Technologies

Disease gene identification by MPS became possible because of three main developments: the technology of sequencing multiple genetic regions simultaneously; the success of the Human Genome Project in providing a complete and reliable reference genome for comparison with the test sequence data; and the availability of large databases of genomic information from healthy individuals, and increasingly in patients with disease, to assist in assessment of variants observed.

The pivotal technological breakthrough for MPS was the development of technologies and platforms for simultaneous sequencing of multiple regions of fragmented DNA or RNA in a single experiment. It is beyond the scope of this paper to provide a comprehensive discussion of these technologies. However, briefly: DNA is fragmented and common adapters are ligated to the fragment ends. The fragments are subsequently amplified by PCR, followed by sequencing-by-synthesis, the common adapters providing uniform starting templates for both the amplification and sequencing reactions for all fragments (more recent technological developments, so-called “third-generation” technologies, involve sequencing without this step of any preceding amplification, improving both accuracy and speed of MPS). Sequencing-by-synthesis involves addition of bases to a growing strand: as each base is added, a signal is generated and “read” by the software, thus generating the sequence of each fragment. The sequence of each fragment is then mapped against the human genome, allowing identification of genetic variants present in the sequenced individual(s).

Large databases of genetic variation (such as The HapMap project, UK10K, 1000genomes, Human Variome Project, and NCBI dbSNP) are used in interpreting sequence data obtained through MPS: identified variants can be characterized as part of the “normal” variability seen in the population, or as novel or rare variants and thus more likely to be pathogenic. Of note, population genetic variability differs among populations of different ethnicities; the sequence data of an individual should be compared against an ethnically-matched reference genome sequence. Once the sequence data have been generated and compared with the appropriate reference human genome, the data can be analyzed empirically based on the observed inheritance and population prevalence of the condition under examination.

Although MPS was developed for whole-genome sequencing (WGS), targeted sequencing proved more cost-effective and efficient initially. Thus, prior to amplification, a library of fragments containing regions of particular interest may be selected (for example, by using probes that anneal to specific regions of interest, allowing their subsequent identification and isolation for PCR amplification and sequencing). Whole exome sequencing (WES) with capture and sequencing limited to gene exons may be particularly suited for rare Mendelian disorders since prior to the advent of MPS 85% of monogenic diseases were predicted to arise from protein-coding mutations [18]—whether this will hold true as more causative mutations are identified is as of yet unknown.

The power of MPS methodology is illustrated by the many causative genetic mutations identified since its advent, especially since they are frequently mapped through sequencing of remarkably few individuals. Indeed, some disease genes have been identified through sequencing of a single proband [2], although confirmation of pathogenicity requires subsequent validation, such as genetic evidence in other affected individuals and/or functional studies of the candidate gene.

### 4.2. Mapping Strategies for Monogenic Diseases Using MPS

The experimental design for mapping a monogenic disease by WES does not necessarily require any prior linkage or association data. Rather it depends on the population frequency of disease, the mode of inheritance (including penetrance), and the presence or absence of consanguinity of the affected individuals. These parameters then determine an appropriately parsimonious experimental design—how many and which individuals should be sequenced and what empirical approach should be adopted for analysis of the sequence data. For example, a rare autosomal recessive condition in a non-consanguineous family is likely to arise from compound heterozygosity; identification of two novel (or very rare) damaging variants in a single gene provides strong evidence of likely causality even if only a single affected individual from the family is sequenced. In contrast, mapping an autosomal recessive condition in a consanguineous family is more difficult. In this circumstance, the disease is more likely to arise from homozygous carriage of a novel (or rare) variant carried by both parents. However, a high number of homozygous rare variants would be expected due to consanguinity anyway; determining which of these is most likely pathogenic can be difficult. For an autosomal dominant condition, the most parsimonious experimental design is to sequence distantly-related affected individuals with a large number of meioses (and, by implication, recombination events) separating the affected cases—with n meioses between the individuals, the chance of a dominant variant segregating with affection status by chance is 1/2^n^. It is also possible to map *de novo* dominant conditions by sequencing unrelated individuals and analyzing the data either for a single variant carried by all affected individuals or for unique variants carried in a common gene by each individual [19]. These last analysis strategies depend crucially upon correct clinical phenotyping of the unrelated affected individuals. The inclusion of phenocopies in the analysis would decrease the success of mapping the causative gene—unless the stringent parameters of analysis are relaxed to allow for their possible presence. For example, one can search for a common shared gene amongst only a proportion of affected individuals rather than requiring a variant to be present in the same gene in every sequenced case. An alternative approach includes pathway analysis (searching for variants in a common pathway amongst individuals with a common phenotype). From a clinical viewpoint, large online databases cataloguing observed variants/mutations in patients with common conditions are also useful in identifying likely disease-causing mutation(s) (as examples, the Leiden Open Variation Database and the Genome Medicine Database of Japan).

### 4.3. MPS Limitations

Like all technologies, MPS has limitations. Good sequencing data depend upon sufficient capture of the causative gene by coverage of sufficient depth of sequencing to call homozygous or heterozygous variants (typically, 10-fold coverage is required for calling a homozygous variant and 15-fold for a heterozygous variant). WES off-the-shelf target platforms vary in their coverage of the “whole exome” [20,21], which may result in a gene of interest failing to be sequenced. For example, we (and others) failed to identify the disease-causing mutation in OI type V despite sequencing several families with the condition; the causative mutation was identified in the 5' UTR of *IFITM5* [22,23], a region not captured with the whole exome capture platform we had employed. Less-than-complete whole exome capture can arise for several additional reasons, including new gene annotation subsequent to platform development and production [24]; a manufacturing decision to target only the main transcript for a gene rather than all known transcripts of a gene; and the technical challenges of capturing GC-rich sequences (which are common in the first exons of many genes [25]). Conversely, there are some regions that amplify excessively: if duplicates are not removed, strand-specific PCR-introduced errors may result, skewing variant allele frequencies with consequent effects on variant detection sensitivity and specificity [26]. Regions of genomic sequence similarity may result in non-specificity of target fragment selection—for example, a probe may anneal not only to the desired target exon but also to an unwanted region of high homology which, when incidentally captured and sequenced, results in apparently novel heterozygous variants at those points of difference between the two selected regions (a phenomenon known as multi-mapping [27]).

However, despite these limitations, faced today with the challenge of identifying a rare gene mutation in a disease like FOP, WES of a small number of FOP patients would likely rapidly reveal the recurrent c.617G>A (R206H) ACVR1 mutation, leading to quick recognition of *ACVR1* as the disease-causing gene. If MPS technologies had been available when the search for the FOP gene began, the answer could have been found in 15 weeks, not 15 years. Certainly, this has proved to be the case in many other skeletal dysplasias where researchers are faced with similar issues of small families afflicted with diseases having a detrimental effect upon reproduction and lifespan [2,4].

### 4.4. Rare Variants as a Cause of Complex Diseases?

Although we focused in this paper on the use of new mapping approaches for Mendelian disorders, MPS has also been used for mapping rare variants that contribute to the heritability of complex diseases. The contributions of rare variation in loci that also harbor common susceptibility alleles, or in genomic regions without common susceptibility alleles, are still the subject of active research. Whilst many examples exist of rare variants contributing to loci harboring common variant associations, these are few by comparison with the total number of common variant associations identified to date. Indeed, targeted sequencing of 25 loci associated with autoimmune disorders in nearly 25,000 individuals with six autoimmune phenotypes and just over 17,000 controls failed to identify any rare variants contributing significantly to immune-mediated disease susceptibility [28]. Styrkarsdottir *et al.* used whole genome scanning in the Icelandic population to identify a rare variant in *LGR4* associated with both bone mineral density and fracture risk [29]. WES identified mutations in *WNT1* as the cause of autosomal dominant early-onset osteoporosis in some families; however, as mutations in *WNT1* were also identified in consanguineous families with autosomal recessive osteogenesis imperfecta it would perhaps be more correct to regard the families diagnosed with AD osteoporosis as having a subtle form of OI and/or a monogenic skeletal dysplasia rather than the common polygenic disease of osteoporosis [30,31]. Studies that conducted dense rare-variant genotyping, such as Immunochip-based analyses of immune-mediated diseases, have had little success in identifying novel rare variant associations, despite large sample sizes [29,32,33].

## 5. Conclusions and Challenges

After mapping a disease-causing gene, many challenges remain, and many of these challenges are likely to be met through the resources and technologies that continue to build on the foundation of the Human Genome Project.

One important consideration is in understanding the multiple genetic contributions to disease onset and progression in addition to the primary causative gene in monogenic disorders. In FOP, for example, although the main disease characteristics are unique and readily recognized, variability in the age of disease onset, the rate of disease progression, and the severity of disease can be high, even in the context of the same ACVR1 c.617G>A mutation. Such phenotypic variability is likely influenced by underlying genetic causes. Identification of genetic modifiers that “protect” an individual with an FOP mutation, for example by directing a late onset or less aggressive disease progression, would provide new therapeutic targets and strategies for treating the disease. Similar issues exist for many common heritable diseases, such as osteogenesis imperfecta and Marfan’s syndrome. With further development of genome technologies, the ability to understand phenotypic variability and the participation of genetic modifiers is becoming a reality.

The ultimate challenge is to elucidate the functions of the target gene and the consequences of its mutant forms, and, most importantly, the translation of this knowledge to treatments. Identification of the specific mutation in *ACVR1* has clear and important diagnostic value, providing a means to confirm suspicion of FOP based on toe malformations or, in cases of potential inheritance (and when sequencing in early life becomes more commonplace), a means to diagnose the condition before irreversible clinical manifestations occur allowing for early intervention. Identification of the target pathway and the specific mutation mechanism in FOP has opened up therapeutic strategies for this disease. Additionally, although the roles of BMP signaling in a wide range of tissue development and homeostasis functions had been well established and the signaling pathway and its components were well defined prior to identifying the FOP mutation, the roles of ACVR1/ALK2 in these processes were unrecognized and poorly understood. FOP identified ACVR1 as a key regulator of skeletal development and bone formation, providing an important new focus for skeletal biology and regenerative medicine. This has been the case for many skeletal dysplasias mapped to date, in which the causative gene was often not known to be involved in bone prior to its identification [34]. There are many examples of such findings providing important insights into musculoskeletal development and pathology, and many current treatments have been developed based on genetic discoveries—for example the development of anti-sclerostin antibodies based on the discovery that the high bone mass conditions of sclerosteosis and van Buchem’s disease arise from mutations in the gene for sclerostin [35,36]. The power of MPS to map disease-associated mutations will thus benefit not only affected individuals and families, but also lead to a dramatic expansion in our understanding of human diseases. This will inform development of new therapies not only for rare monogenic disorders but also for diseases common in the general population.
